# In-Person and Remote Workshops for People With Neurocognitive Disorders: Recommendations From a Delphi Panel

**DOI:** 10.3389/fnagi.2021.747804

**Published:** 2022-01-21

**Authors:** Valeria Manera, Luis Agüera-Ortiz, Florence Askenazy, Bruno Dubois, Xavier Corveleyn, Liam Cross, Emma Febvre-Richards, Roxane Fabre, Nathalie Fernandez, Pierre Foulon, Auriane Gros, Cedric Gueyraud, Mikael Lebourhis, Patrick Malléa, Léa Martinez, Marie-Pierre Pancrazi, Magali Payne, Vincent Robert, Laurent Tamagno, Susanne Thümmler, Philippe Robert

**Affiliations:** ^1^Cognition Behaviour Technology (CoBTeK) Lab, Université Côte d’Azur, Nice, France; ^2^Servicio de Psiquiatría, Instituto de Investigación (i + 12), Hospital Universitario 12 de Octubre, Madrid, Spain; ^3^Centro de Investigación Biomédica en Red de Salud Mental (CIBERSAM), Madrid, Spain; ^4^University Department of Child and Adolescent Psychiatry, Children’s Hospitals of Nice CHU-Lenval, Nice, France; ^5^Institut de la mémoire et de la Maladie d’Alzheimer (IM2A), INSERM, U1127, AP-HP, Sorbonne Université, Hôpital de la Pitié-Salpêtrière, Paris, France; ^6^Institut du Cerveau et de la Moelle Épinière (ICM), INSERM, U1127, AP-HP, Sorbonne Université, Hôpital de la Pitié-Salpêtrière, Paris, France; ^7^Laboratoire d’Anthropologie et de Psychologie Cliniques, Cognitives et Sociales (LAPCOS), Université Côte d’Azur, Nice, France; ^8^Maison des Sciences de l’Homme et de la Société Sud-Est (MSHS Sud-Est), Nice, France; ^9^Department of Psychology, Edge Hill University, Liverpool, United Kingdom; ^10^Whiti o Rehua School of Art, College of Creative Arts, Massey University, Wellington, New Zealand; ^11^Public Health Department, Nice University Hospital, Côte d’Azur University, Nice, France; ^12^Accueil de Jour Fondation GSF Jean-Louis Noisiez, Biot, France; ^13^GENIOUS Healthcare–Mindmaze Group Co., Lausanne, Switzerland; ^14^Centre National de Formation aux Métiers du Jeu et du Jouet (FM2J), Caluire-et-Cuire, France; ^15^Asmodee Research, Asmodee, Guyancourt, France; ^16^NEHS Digital Co., Paris, France; ^17^Centre de Recherches sur la Cognition et l’Apprentissage, Université de Poitiers, Poitiers, France; ^18^Centre Hospitalier de Bastia, Bastia, France; ^19^Centre Hospitalier Universitaire de Nice, Service Clinique Gériatrique du Cerveau et du Mouvement, Université Côte d’Azur, Nice, France; ^20^Centre Mémoire de Ressources et de Recherche, Université Côte d’Azur, Nice, France; ^21^Association Innovation Alzheimer, Nice, France; ^22^MAMAC Museum, Nice, France

**Keywords:** remote/hybrid workshop, neurocognitive disorders, recommendations (guidelines), arts, board game, workshop

## Abstract

Workshops using arts and board games are forms of non-pharmacological intervention widely employed in seniors with neurocognitive disorders. However, clear guidelines on how to conduct these workshops are missing. The objective of the Art and Game project (AGAP) was to draft recommendations on the structure and content of workshops for elderly people with neurocognitive disorders and healthy seniors, with a particular focus on remote/hybrid workshops, in which at least a part of the participants is connected remotely. Recommendations were gathered using a Delphi methodology. The expert panel (*N* = 18) included experts in the health, art and/or board games domains. They answered questions via two rounds of web-surveys, and then discussed the results in a plenary meeting. Some of the questions were also shared with the general public (*N* = 101). Both the experts and the general public suggested that organizing workshops in a hybrid format (some face-to-face sessions, some virtual session) is feasible and interesting for people with neurocognitive disorders. We reported guidelines on the overall structure of workshops, practical tips on how to organize remote workshops, and a SWOT analysis of the use of remote/hybrid workshops. The guidelines may be employed by clinicians to decide, based on their needs and constraints, what interventions and what kind of workshop format to employ, as well as by researcher to standardize procedures to assess the effectiveness of non-pharmacological treatments for people with neurocognitive disorders.

## Introduction

A workshop is an activity allowing several individuals to work together and share around an activity, a topic. Workshops concern all generations, from children to seniors. Workshops are used in different domains to promote education using interactive, sometimes ludic formats, focused on achieving practical individual or group objectives. Similarly, they are at the basis of all co-design approaches, in which participants are asked to generate ideas to find a solution to a problem or to brainstorm about a topic (Brown, 2008; Hamidi et al., 2014; Yock et al., 2015). In the field of health, and in particular in the domain of mental health, the practice of workshops has been around for a long time at the level of treatment (e.g., occupational therapy, reminiscence therapy, ergotherapy) but also at the level of prevention (e.g., memory workshop). As such, workshops can be included in the vast field of psychosocial, non-pharmacological interventions. These interventions focus on psychological or social factors, can improve symptoms, functioning, quality of life and more globally aim to prevent, treat, or cure a health problem (Barbui et al., 2020). It takes the form of a product, a method, a program or a service, whose content is known by the user (Ninot et al., 2017). Non-pharmacological intervention’s implementations require relational, communicational, and ethical skills. Ideally, non-pharmacological interventions effects need to be explained by biological, cognitive, behavioral, and social processes and are the subjects of efficacy studies. This is important because the effectiveness of non-pharmacological interventions cannot be taken for granted even if they have been known and used for “the dawn of time.” Setting up a clinical study does therefore require using the same scientifically sound methods as for pharmacological interventions but with the adaptation required in the context of non-pharmacological intervention which are very heterogeneous. It has been highlighted that there is a lack of precise description of the non-pharmacological interventions in a consecutive sample of randomized trials, thus making reproducibility hard (Hoffmann et al., 2013).

**FIGURE 1:**
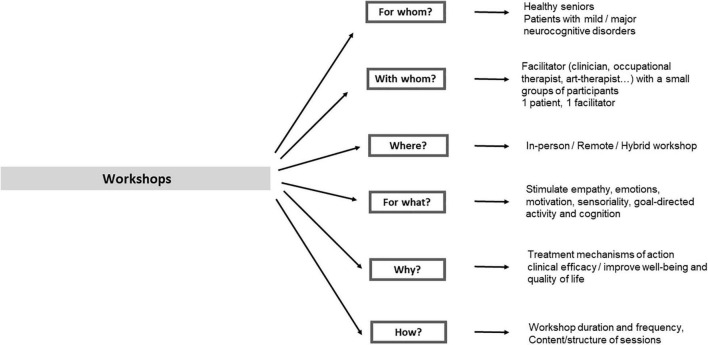
Main parameters defining the features of a workshop session.

Non-pharmacological interventions have been the subject of great interest for many decades among healthcare professionals involved in the prevention of cognitive disorders in the elderly and treatment of Alzheimer’s disease and related disorders (Moniz-Cook et al., 2008). Gradually, clinical research has been carried out to demonstrate the effectiveness of this type of interventions (Cammisuli et al., 2016), with the aim to provide scientific evidence to validate their use in daily clinical practice. For neurocognitive disorders, Alzheimer’s disease and related disorders at different stages, the proliferation of studies has led to several literature reviews assessing the overall efficacy and safety of different non-pharmacological interventions (e.g., Lee et al., 2013). A key of success for treatment efficacy is clearly personalization: more individualized treatments that take into account the patient’s past preferences and environmental factors can improve treatment outcomes, for instance in the case of apathy (Theleritis et al., 2018). Most of these systematic reviews have identified possible benefits of non-pharmacological interventions, but the conclusions are often quite similar, and points out to the need of more controlled and well-designed studies to precisely define the outcome measures (e.g., cognition, behavioral symptoms, well-being) but also the content of non-pharmacological interventions (which intervention?) and the format and context (e.g., what frequency? What duration? With whom? Where? see Figure 1; Teixeira et al., 2012; Wang et al., 2014; Rodakowski et al., 2015; Couch et al., 2020; Yao et al., 2020) which can insure that successful training can be precisely reproduced.

The first objective of the Art and Game project (AGAP), initiated by the CoBTeK lab of Université Cote d’Azur (Nice, France) was to draft recommendations on the structure of workshops for elderly people with neurocognitive disorders and cognitively healthy seniors. In this project we focused on two main areas, namely arts and board games, which are both consistently used in these populations. The main objectives of workshops using arts are to improve well-being, but also engage, stimulate, and relax (Newman, 2003; Davies and Duff, 2005; Johnson et al., 2017; Butler et al., 2018; Windle et al., 2018). Uniting touch, breath, and vision to create a focus, meditation and “flow” that is comparable to the principles of mindfulness, responding directly to the “here and now” (Paller et al., 2015; Berk et al., 2018; Chan et al., 2020). Board games are mainly used to stimulate cognitive activities, increase motivation and positive emotions, and promote social interactions in people with neurocognitive disorders. They may help slowing down cognitive decline and reduce depression in these populations (Dartigues et al., 2013; Zhao et al., 2016; Ma et al., 2018). Despite the clear differences in terms of type of activities, objectives, and methods, we believe that the overall structure of workshops using arts and board-games can have many commonalities, which we wanted to highlight. Indeed, both include a wide range of activities and subtypes, and target not only on cognition, but also on emotions, motivation, and well-being.

The second objective of AGAP project came from what we experienced during the COVID crisis, where many health professionals were struggling with the new paradigm. In-person workshops were the norm for decades. Most of the methods and technical tools that are available today were developed to support in-person workshops. With the rapid development of remote teaching,^1^ assessment (König et al., 2021), and interventions proposed by health insurances.^2^ The role of new information and communication technologies (ICT) in this context became suddenly prominent, and it was rapidly recognized that the classical workshop and teaching formats were not completely pertinent to remote use and needed adaptations. Starting form this experience and recognizing the lack of guidelines for workshops in this domain, we decided to organize an expert meeting aimed to provide recommendation for the format and content of remote workshops, with a specific focus on workshops using arts and board games.

## Materials and Methods

The expert panel included 18 professionals, 10 coming from the health domain (researchers and healthcare professionals), 4 from the game domain (game industry manager), and 4 from the arts domain (artists). Among them, 4 (23.5%) reported to be experts (“Jedi”) in board games, 4 (38.9%) in arts, and 12 (70.6%) in health. Following a four-step Delphi methodology (Linstone and Turoff, 1975), the recommendations were developed in a four-step process: after a literature review and an initial validation of the relevant topics to be treated, the experts were asked to respond to questions in two rounds of web-surveys. After each round, a facilitator (PR) provides a summary of the experts’ responses and encourages the experts to analyze, comment, and (eventually) revise their earlier answers in light of the commentaries of other members of the panel. The results were discussed in a final consensus meeting. Some of the questions employed in the first web-survey were also shared across the public interested in arts and/or games in the context of thematic research projects, such as Art&Santé^3^ or Game in lab projects.^4^ Responses were obtained from 101 volunteers (64 females and 37 males; 49 aged below 60 years, 62 aged 60 years or more). In terms of socio-professional category, 40.6% (*N* = 41) were retired, 24.8% (*N* = 25) were working in a clinical or social domain, 18.9% (*N* = 19) in the education domain (students, teachers, or researchers), and 15.8% (*N* = 16) in the industry domain. 26.3% (*N* = 26) reported to be experts (“*Jedi*”) in games, 24.2% (*N* = 24) reported to be experts in arts, while 50.4% (*N* = 50) reported to be experts in the domain of health. No statistical difference in the proportion of *Jedi* in the different domains was found between experts and general public.

### Web-Surveys

#### Expert Group

The experts were asked to answer questions via web-surveys in two rounds (between February and April 2021) using Google Forms. After each round, a facilitator (PR) provided a summary of the experts’ responses, and encouraged the experts to analyze, comment and (eventually) revise their earlier responses considering the commentaries of other members of the panel. Questions in the two rounds included rating questions, yes-no questions, and open question, divided in different domains: (a) general questions on the objectives and use of *workshops* in elderly people with and without cognitive impairment; (b) questions on workshops in the domain of arts; (c) questions on workshops in the domain of games, and (d) questions on the feasibility of organizing workshops remotely or using a hybrid format. The rating and yes-no questions employed in Delphi 1 and 2 in these domains are reported in Table 1 and Figure 1. In addition, the experts were asked (e) questions regarding the practical aspects of workshops organization (number of participants, duration, and frequency of sessions), as reported in Table 2, and to rate the pertinence of using different types of arts and games to stimulate different cognitive functions, disorders of emotions and motivation, and physical activity, as reported in Table 3.

**TABLE 1:** Delphi round 1 and 2 rating and yes-no questions (general questions/questions workshops using art and workshops using board games.

		Experts (*N* = 18)	General public * (*N* = 101)
**General questions**		Median, IQR/5**	
1a. How important are non-pharmacological approaches to improve mental health in			
	Seniors with MILD neurocognitive disorders?	5.0 (0.8)	
	Seniors with MAJOR neurocognitive disorders?	5.0 (0.0)	
	Healthy seniors?	5.0 (0.8)	
		*N* (%***) of Yes	*N* (%***) of Yes
2a. Whatever the theme and the type of activity offered during a workshop, the workshop method must			
	Facilitate social interactions between participants and with the facilitator	17 (100%)	
	Offer a rewarding experience for each participant and in at the same time advance the work of the group	16 (100%)	
	Use an underlying theory. For example: Facilitate the creation of a transitional space in Winnicot’s sense	10 (83%)	
3a. Among the overall objectives of workshops (whatever the theme, or the mediation tool) we can list			
	Stimulate empathy and spontaneous emotions, in reaction to the group environment	14 (93%)	94 (100%)
	Stimulate motivation in goal-directed behaviors and cognitive activity	16 (94%)	95 (98%)
	Stimulate well-being, improve quality of life	14 (100%)	94 (98%)
	Stimulate sensoriality	14 (93%)	93 (97%)
4a. Feedback to participants at the end of a workshop can take the form of			
	Sending a video showing the recording of one or more sessions	11 (85%)	55 (70%)
	Sending a report, or a photographic composition	16 (100%)	79 (90%)
	Sending a catalog	9 (82%)	32 (54%)
	Invitation to a virtual or real exhibition	17 (100%)	83 (93%)
**Workshops using arts**		Median, IQR/5**	
1b. Can you rate the following objectives for workshops using arts?			
	Stimulate goal-directed behaviors	3.0 (1.0)	89 (98%)
	Stimulate cognitive activity	3.0 (1.0)	81 (92%)
	Stimulate emotions	4.5 (1.0)	95 (98%)
	Stimulate social interactions	3.5 (1.0)	86 (98%)
	Stimulate sensoriality	4.0 (2.0)	93 (100%)
		*N* (%***) of Yes	*N* (%***) of Yes
2b. Can the workshops using arts be used for individual practice (1 patient, 1 facilitator)?		15 (94%)	
3b. Can workshops using board games stimulate sensoriality?			
	Earing	14 (100%)	
	Vision	14 (100%)	
	Praxis	15 (100%)	
	Olfaction	12 (100%)	
4b. Do we need a specific definition of therapeutic workshops using art?		10 (67%)	
**Workshops using board games**		Median, IQR/5**	
1c. Can you rate the following objectives for workshops using board games?			
	Stimulate goal-directed behaviors	4.0 (1.0)	91 (100%)
	Stimulate cognitive activity	4.0 (0.0)	96 (99%)
	Stimulate emotions	3.0 (1.0)	84 (94%)
	Stimulate social interactions	4.0 (1.0)	96 (98%)
	Stimulate sensoriality	3.0 (1.0)	55 (71%)
		*N* (%***) of Yes	*N* (%***) of Yes
2c. Can the workshops using board be used for individual practice (1 patient, 1 facilitator)?		17 (94%)	
3c. Can workshops using board games stimulate sensoriality?			
	Earing	13 (100%)	
	Vision	14 (100%)	
	Praxis	15 (100%)	
	Olfaction	11 (91%)	
4c. Do we need a specific definition of therapeutic workshops using board games?		11 (79%)	
Workshops organized remotely and/or with a hybrid format		*N* (%*) of Yes	*N* (%*) of Yes
1d. Can a session (several workshops) be done in a hybrid format? (some face-to-face sessions and some virtual sessions)?		18 (100%)	78 (90%)
2d. A workshop session can be done face to face with the facilitator for some participants, and remotely for some others?		14 (82%)	65 (72%)
3d. Is it possible to animate workshops using art in a virtual way?		15 (100%)	60 (67%)
4d. Is it possible to animate workshops using board games in a virtual way?		18 (100%)	56 (67%)

* The general public responded to a selection of question.

** Five-point rating scale: 1 = Not important/pertinent at all; 2 = Not very important/pertinent; 3 = Important/Pertinent; 4 = Very important/pertinent; 5 = Extremely important/pertinent.

*** Number and percentage of participants that responded Yes. Percentages are calculated based on the number of people who responded “Yes” or “No” (“I don’t know–Prefer not to answer” responses were not included).

**TABLE 2:** Delphi round 1 and 2 questions for the experts on the organizational aspects of workshops.

In a workshop intervention		*N* = 18	General public (*N* = 101)
1e. How many sessions per week should be performed (from 1 to 5)?		Median	Median
	Healthy seniors (prevention)	2	
	Patients with MILD neurocognitive disorders?	2	
	Patients with MAJOR neurocognitive disorders?	2.5	
2e. How many weeks should the intervention last (between less than 3 weeks and more than 12 weeks)?			
	Healthy seniors (prevention)	6–12 weeks	
	Patients with MILD neurocognitive disorders?	6–12 weeks	
	Patients with MAJOR neurocognitive disorders?	6–12 weeks	
3e. How long should each session last (from less than 15-min to more than 1 h)?			
	Healthy seniors (prevention)	30–60 min	
	Patients with MILD neurocognitive disorders?	30–60 min	
	Patients with MAJOR neurocognitive disorders?	15–30 min	
4e. If we take a 45-min session, how much time should be devoted to			
	Group discussion	10 min	
	Activity (art creation, board games)	25 min	
	Listening to the facilitator	5–10 min	
5e. How many participants can be involved in a workshop session using art remotely?		7–8 participants	5–6 participants
6e. How many participants can be involved in a workshop session using games remotely?		6 participants	5–6 participants
			

**TABLE 3:** Mean ratings on the interest of using different types of arts and games for specific disorders.

	Plastic arts		Photography		Music		Cinema		Writing		Social games		Mind games		Dexterity games		Memory games	
	mean	SD	mean	SD	mean	SD	mean	SD	mean	SD	mean	SD	mean	SD	mean	SD	mean	SD
Attention	**4,1**	**1,0**	4,0	1,1	**4,2**	**1,0**	3,6	1,0	3,4	1,2	3,4	1,4	3,8	1,2	3,9	1,3	4,0	1,2
Memory	3,7	1,0	4,1	0,9	**4,3**	**0,9**	3,9	0,8	3,5	1,1	3,8	1,2	3,8	1,1	3,6	1,2	**4,4**	**0,7**
Executive functions	**4,3**	**0,7**	3,8	0,9	3,6	1,1	3,4	1,1	4,0	1,1	**4,1**	**1,0**	**4,1**	**1,0**	**4,1**	**0,9**	3,6	0,9
Depression	**4,6**	**0,6**	4,5	0,7	**4,8**	**0,4**	4,3	1,1	3,9	1,2	4,1	1,2	3,2	1,3	3,4	1,3	3,4	1,2
Agitation	**4,2**	**1,0**	3,4	1,4	**4,7**	**0,7**	3,6	1,3	3,0	1,1	3,4	1,2	3,1	1,3	3,5	1,1	3,0	1,3
Interests	**4,5**	**0,8**	**4,3**	**0,8**	**4,5**	**0,7**	4,2	1,2	3,5	1,2	4,1	0,8	3,5	1,1	3,8	1,1	3,5	1,0
Social interaction	4,0	1,0	3,8	0,9	4,0	1,2	**4,2**	**1,0**	3,3	1,2	**4,7**	**0,5**	3,6	1,4	3,7	1,2	3,7	1,2
Physical activity	**3,8**	**0,9**	3,5	1,0	**3,9**	**1,2**	**3,8**	**0,9**	3,2	1,0	3,6	1,1	3,3	1,1	**3,9**	**1,2**	3,1	1,1

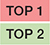

*1: Not pertinent at all, 5: Completely pertinent.*

Rating questions employed a five-point Likert scale (1 = Not important/pertinent at all; 2 = Not very important/pertinent; 3 = Important/Pertinent; 4 = Very important/pertinent; 5 = Extremely important/pertinent). After each rating question, participants could provide open comments. The open questions for the experts in Delphi 1 round included: comments on the definition of workshop (the responses were employed to improve the definition provided in the introduction); and providing a list of maximum three board games and arts that may be useful during workshops (the listed examples were employed in Delphi 2 round, see Table 3). Open questions in Delphi 2 round included providing suggestions to facilitate the activity of participants connected remotely; and ideas on how to promote stimulation of emotions, cognitive activity, social interactions and sensoriality using arts and board games in remote sessions. After round 2, a first draft of the recommendations was circulated among the experts.

#### General Public

The web-survey circulated among the public included a selection of the questions asked to the experts, including questions on the notion of workshop and its use in the arts and games domains, and questions on feasibility of organizing workshops remotely or using a hybrid format. All these questions are listed in Table 1. The survey was circulated between March and April 2021.

### Final Consensus Meeting

The two web-surveys’ results and the open discussion points were revised by the task force during a hybrid plenary meeting held on June 4, 2021, in Nice (France). Six experts were physically present in Nice, while 12 were connected remotely. Beyond presenting a summary of the two web-surveys, the experts were proposed (through Zoom survey tool) with a list of suggestions on how to facilitate remote-hybrid sessions (derived from open suggestions provided in the Delphi 2 round) and asked if they agreed (yes or no) to include each statement in the final recommendations. The questionnaire included questions concerning organizational aspects of workshops, concerning workshop preparation and specific questions for workshops using arts, and workshops using games. The items that reached a consensus of at least 80% are reported in Table 4.

**TABLE 4:** Recommendations on how to facilitate the activity of the participants connected remotely in remote/hybrid workshops.

Organization
Reduce the sessions duration compared to classical workshops
Ask regularly questions about satisfaction and provide feedback on the sessions
Regulate turn taking and precisely define the activities
Promote the use of group and private chats to help participants when they need
**Preparation**
Ask participants to do some “homework” to be discussed during the sessions
Invite caregivers to join the sessions (if needed) to facilitate patients’ participation
Contact participants before the session to ensure that that they are able to connect and they have the required materials/setup
Send/ship tools and materials before the session
**Concerning workshops using ARTS to stimulate emotions and sensoriality**
Employ a variety of multi-media materials (music, video, sounds, images, etc.), and ensure a good quality of rendering (e.g., big screen, good microphones and speakers)
Ask direct feedback about their feelings and emotions, and ask to share personal memories and interests
Ask to collect specific materials that we can touch, smell. before the session
Use mental imagery to stimulate senses that cannot be directly stimulated (smell)
Promote group activities in which participants build something together (e.g., every participants build a piece of a global artwork)
**Concerning workshops using BOARD GAMES to stimulate social interactions and cognitive activity**
Suggest participants (if they want) to meet outside de sessions
Increase the number of social exchanges during the sessions, and of group/couple activities (multi-role games);
Include group discussions and feedback before and/or after the game activity
Create a group dynamic (e.g., asking each participant to select a personalized avatar that can evolve over time)

## Results

The results of the rating questions (median and interquartile range, IQR) and the Yes-No questions (number of “Yes” responses, and percentage of “Yes” responses calculated on the number of people who responded “Yes” or “No”; “I don’t know–Prefer not to answer” responses were not considered) are reported in Table 1. The first column contains the experts’ responses, and the second column the general public’s responses. For each question, we compared the percentage of “Yes” responses between the experts and the members of the general public using Chi^2^ or Ficher tests. No significant difference was found, thus suggesting that the opinion of the general public converged with that of the experts. For the general public, we also compared the percentage of “Yes” responses provided by those who reported to be experts (Jedi) in arts and/or games and those who reported to be novices (Padawan) using Chi^2^ tests. No significant difference was found, thus suggesting that the novices and the experts in the general public had a similar opinion in all the investigated topics.

### General Questions

Results of the two web-surveys are reported in Table 1. The experts reported that (Q1a) non-pharmacological approaches to improve mental health are “extremely important” for healthy seniors, as well as for patients with mild and major neurocognitive disorders. The totality of experts acknowledged that (Q2a) workshops should facilitate social interactions between participants and with the facilitator, and offer a rewarding experience for each participant and in at the same time advance the work of the group. 83% of the experts suggested that it is important for workshops to use an underlying theory [for example: facilitate the creation of a transitional space in Winnicot’s sense (Winnicott, 1975)]. Other listed theories that can be used to design a workshop are Biodesign (Yock et al., 2015), Design Thinking (Brown, 2008), Co-design (Hamidi et al., 2014), the Community of Practices (Wenger, 2011), and participatory action methods (Serrel, 1998). Among the overall objectives of workshops (Q3a), experts and responders from the general public agreed on the importance of: stimulating empathy and spontaneous emotions in reaction to the group environment; stimulating motivation in goal-directed behaviors and cognitive activity; stimulating well-being, improve quality of life; and stimulating sensoriality. In terms of (Q4a) feedback provided to the participants at the end of a workshop series, for both the experts and the members of the general public the most popular feedback was the invitation to a virtual or real exhibition or sending a report/a photographic composition. 85% of the experts and 70% of the members of the general public agreed on sending a video showing the recording of one or more sessions (with attention devoted to privacy issues; only participants that agreed to appear in the video should be visible). Sending a catalog was considered as possible feedback by 82% of the experts, and by 54% of the members of the members of the general public.

### Questions on Workshops Using Arts

Results are reported in Table 1. In terms of the objectives of workshops using arts (Q1b), the experts rated as “important” stimulating goal directed behavior and cognitive activity, as between “important” and “very important” stimulating social interactions, as “very important” to stimulate sensoriality, and as between “very important” and “extremely important” stimulating emotions. So, stimulating emotions and sensoriality seemed to be the top two rated objectives.

**FIGURE 2:**
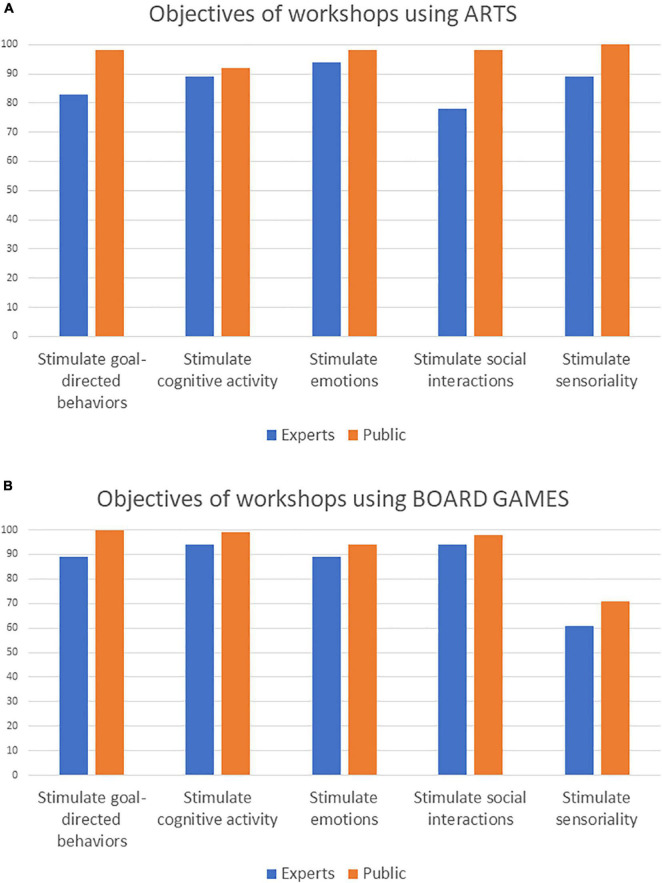
Percentage of experts (*N* = 18) and general public (*N* = 101) agreeing on the objectives of workshops using **(A)** arts and **(B)** board-games.

More than 90% of the members of the general public agreed on the importance of all these objectives. 94% of the experts agreed that (Q2b) workshops using arts be used for individual practice (1 patient, 1 facilitator), and the totality of experts that expressed an opinion agreed that (Q3b) they can stimulate sensoriality (earing, vision, praxis, and olfaction). Only 67% of the experts agreed that (Q4b) there is need for a specific definition of therapeutic workshops using arts. In order to visually compare the opinion of the experts and the general public, we re-coded the expert responses in “yes” (“important,” “very important” and “extremely important” responses’) and “no” (“not very important” and “not important at all”). Results are reported in Figure 2A.

### Questions on Workshops Using Board Games

Results are reported in Table 1. In terms of the objectives of workshops using board games (Q1c), the experts rated as “important” stimulating emotions (94% of the member of the general public agreed on its importance) and sensoriality (71% of the members of the general public agreed on its importance), and as “very important” stimulating goal-directed behaviors, cognitive activity and social interaction (more than 95% of the members of the general public agreed on the importance of these three objectives). So, the top-two objectives were stimulating cognitive activity and social interaction. 94% of the experts agreed that (Q2c) workshops using board games can be used for individual practice (1 patient, 1 facilitator), and the totality of experts that expressed an opinion agreed that (Q3c) they can stimulate sensoriality (earing, vision, and praxis), and 91% agreed that board games can be employed to stimulate olfaction. 79% of the experts agreed that (Q4c) there is need for a specific definition of therapeutic workshops using board games. In order to visually compare the opinion of the experts and the general public, we re-coded the expert responses in “yes” (“important,” “very important” and “extremely important” responses’) and “no” (“not very important” and “not important at all”). Results are reported in Figure 2B.

### How to Select Arts and Board Games Based on the Patients’ Disorders

The experts were asked to rate of a five-point Likert scale how much different types of arts and board games were relevant to use to stimulate specific abilities and train specific disorders. Results (mean and standard deviation) are reported in Table 3 (the top 2 arts/board games for each category are highlighted). The experts suggested that music and plastic arts are the most relevant to train attention; memory games and music were rated as the most adapted to train memory, while plastic arts and games (social games, mind games, and dexterity games) were the best adapted to train executive functions. In terms of neuropsychiatric symptoms, music and plastic arts were rated as the most relevant to cope with depression and agitation. To stimulate interests, the most relevant activities were music, plastic arts, and photography. Social games and cinema were rated as the most relevant to stimulate social interaction, while to stimulate physical activity the expert suggested as the most adapted activities dexterity games, music, cinema and plastic arts. These ratings may help clinicians to select the most relevant activity to propose to the patient based on his/her profile and interests (and based on the options available).

### Workshops Organized Remotely/Using a Hybrid Format

Results obtained in the web-surveys are reported in Table 1. All the experts and 90% of the members of the general public agreed that (Q1d) a session (several workshops) can be done in a hybrid format, with some face-to-face sessions and some virtual sessions. 82% of the experts and 72% of the members of the general public agreed on the fact that (Q2d) a workshop session can be done face to face with the facilitator for some participants, and remotely for some others. Concerning workshops specifically focused on arts (Q3d) and board games (Q4d), all the experts agreed that these workshops can be organized virtually, while only 67% of the members of the general public that expressed an opinion agreed on the virtual format. During the expert meeting, the experts agreed on the interest to include some recommendations on how to organize remote/hybrid workshops. All the 17 proposed statements reached more than 80% of agreement and are thus reported in Table 4.

### Workshop Organization

In terms of practical organization, as reported in Table 2, the experts suggested that (Q1e) two sessions per week is the optimal session frequency for healthy seniors (prevention; IQR: 2) and people with mild neurocognitive disorders (IQR: 1), while the median score was between 2 and 3 sessions per week (IQR: 1) for people with major neurocognitive disorders. In terms of total duration (Q2e), the median score was 6–12 weeks for both healthy seniors and people with mild and major neurocognitive disorders. The optimal session duration (Q3e) was between 30 and 60 min for healthy people and people with mild neurocognitive disorders, while 15–30 min was selected as the most relevant for people with major neurocognitive disorders. Considering a 45-min session (Q4e), the experts suggested (median scores) that 10 min should be devoted to group discussion (IQR: 0), 25 min to the activity (arts or board games; IQR: 10 min) and 5–10 min to listening to the facilitator (IQR: 5–10 min). When conducting workshops remotely, the experts suggested that the size of the participants’ group should be of 7–8 people (IQR: 2) for workshops using arts (Q5e), and around 6 participants (IQR: 4) for workshops using board games (Q6e). The members of the general public suggested to involve 5–6 participants for workshops using arts and board games. All these recommendations (Table 3) were implemented in the application AT@OREVAS,^5^ which can be employed by clinicians and therapists to plan the ideal workshop format based on their clinical objectives and needs.

## Discussion

Workshops based on arts and board games are prominent examples of non-pharmacological interventions that are consistently employed in the elderly population (Dartigues et al., 2013; Huhtinen-Hildén, 2014), and have many commonalities in terms of format and objectives. As stated by the World Health Organization,^6^ globally the population is aging rapidly. Between 2015 and 2050, the proportion of the world’s population over 60 years will nearly double, from 12 to 22%. Prompt recognition and treatment of mental, neurological and substance use disorders in older adults is essential. Both psychosocial interventions and pharmacological solutions are recommended. In this publication, we present recommendations drafted during an international consensus group concerning the contents, objectives and format of workshops using arts and board games for the elderlies. Given that the recommendations were drafted during the COVID-19 pandemic and the consequent social distancing requirements, special attention was devoted to draft recommendations for workshops using a remote and/or hybrid format (some participants connected remotely, other physically present; and/or some sessions conducted remotely, some others in in-person format).

### For Whom?

The experts acknowledged that workshops are extremely important and useful both for healthy elderly people (for prevention purposes, but also to improve well-being and quality of life) and for people with mild and major neurocognitive disorders.

### For What?

Workshops should facilitate social interactions between participants and with the facilitator, and offer a rewarding experience for each participant, and in at the same time advance the work of the group. The overall objectives of workshops include stimulating empathy, emotions, motivation, sensoriality, goal-directed activity and cognition, with the final aim of improving quality of life and well-being. These overall objectives are common to workshops using arts and board games. However, these two categories of workshops have also some specificities in terms of objectives. Converging with previous recommendations on art-therapy for people with Alzheimer’s Disease and related disorders,^7^ based on the experts’ opinion, workshops using arts seems to be particularly suitable to stimulate emotions and sensoriality, but can also be useful to reduce behavioral symptoms, and stimulate goal-directed activity and cognition. As reported in Table 3, different types of arts may be relevant to stimulate different aspects. For instance, music and plastic arts are very suitable to improve behavioral symptoms such as depression and agitation, to stimulate interests and physical activity, as well as to train attention and executive functions. Cinema is the most useful to stimulate social interactions, and photography is well suited to stimulate interests. Workshops using board-games are ideal to favor social interactions (especially social games) and stimulate goal-directed behaviors and cognitive activity. Memory games are ideal to stimulate memory, while if the target is executive functions, clinicians should better employ mind games, dexterity games or social games. Dexterity games are also very useful to train physical activity. As personalization is a key element for success of non-pharmacological interventions, special attention should be devoted to the identification of previous interests and preferences of the group members, in order maximize treatment efficacy (Theleritis et al., 2018; Starkstein and Hayhow, 2019). In addition, attention should be paid to the type of deficit affecting the patients (cognitive, neuropsychiatric, and/or sensory), as interventions should be tailored to these deficits. For instance, someone with untreated auditory problems may find hard to follow a workshop based on music, especially remotely.

### With Whom?

The classical format of workshops is represented by a group of participants and a facilitator (usually a clinician, such as an occupational therapist, a psychologist, a speech therapist, a nurse, etc.). For remote workshops, no more than 7–8 participants should be involved. The experts suggested that the main format of group workshops may be also employed for individual sessions (one participant and one facilitator), that could be chosen when patients do not work well in a group or need constant help.

### How?

The experts suggested that the ideal workshop duration for elderly people with and without neurocognitive disorders is between 6 and 12 weeks. For healthy seniors and patients with mild neurocognitive disorders, it has been proposed an average of two sessions per week, with a session duration of 30–60 min. For people with major neurocognitive disorders, sessions may be more frequent (2–3 times per week) but shorter (15–30 min). For both workshops using arts and board games, a 45-min session should include 25 min devoted to the main activity (arts or games), a 10-min discussion, and 5–10 min of listening to the facilitator.

### Where?

Due to logistic reasons and other constraints (health related or not), presential workshops are not always possible. The expert group suggested that it is possible to animate workshops using art and board games in a virtual way. A workshop (several sessions) may be organized in a hybrid format, with some face-to-face sessions and some virtual sessions, and face to face with the facilitator for some participants, and remotely for some others. The members of the general public agreed on the interest of employing a hybrid format, with some face-to-face sessions and some virtual sessions, but were more skeptical on the possibility to organize workshops on arts and games completely virtually. This may be due to the fact that the general public finds remote workshops based on arts and board games less engaging/effective, and/or by the additional constraints imposed by the use of digital technologies (e.g., doing arts requires specific materials, not always available at home). In response to this skepticism, the experts proposed some guidelines to favor the realization of remote/hybrid sessions (see Table 4). In terms of workshop organization, remote sessions should be shorter compared to classical workshops (as attention is less focused), the program should be planned more in detail, turn taking should be regulated more precisely, and individual and group feedback should be asked more regularly to adjust the sessions according to the participants’ input (as non-verbal, implicit indicators of participants’ satisfaction are harder to capture remotely). In terms of workshop preparation, it is important to make sure (organizing for instance technical meetings before the workshop) that participants can connect, that they have the necessary equipment (e.g., connected webcam) and setup. If this is not the case, shipping materials and or asking the help of caregiver may be important to ensure a successful participation. Concerning workshops using arts to stimulate emotions and sensoriality, it is important to employ a variety of multi-media materials (music, video, sounds, images, etc.), and ensure a good quality of rendering (e.g., big screen, good microphones and speakers). Materials may be collected/shipped before the sessions (that participants can touch, smell), and mental imagery could be used to stimulate senses that cannot be directly stimulated (e.g., smell). Especially in the context of remote workshops, it would be important that sensory impairments (e.g., hearing problems) are well-compensated, to be able to completely profit from the interventions. Indeed, as suggested by the SENSE-Cog project,^8^ optimizing hearing and vision function is important in improving a range of outcomes for elderly people, especially those with neurocognitive disorders (Hooper et al., 2019; Leroi et al., 2020a,b).

Concerning workshops using board games to stimulate social interactions and cognitive activity, it may be useful to suggest participants (if they want) to meet outside de sessions, and increase the number of social exchanges during the sessions, and of group/couple activities (multi-role games), as well as to create a group dynamic.

### Strengths, Weaknesses, Opportunities, and Threats Analysis on the Use of Remote/Hybrid Workshops

The COVID-19 pandemic suddenly showed the importance of providing recommendations to conduct workshops remotely, to avoid social isolation and interrupting all workshop activities. These recommendations may be useful also outside the pandemic crisis. The experts provided several suggestions on the use of remote workshops, that were used to formulate a Strengths, Weaknesses, Opportunities, and Threats (SWOT) analysis (see Table 5).

**TABLE 5:** Summary of a Strengths, Weakness, Opportunities, and Threats (SWOT) Analysis of using ICT for workshop using art/board games.

Strengths	Weakness
- Can be used to connect people living remotely - Can be used when physical meetings are impossible - Useful for long workshop duration, allowing to extend patient activity at home - Cost/time effectiveness (e.g., no need to commute) - Can be used to facilitate interactions with the whole group, but also between couples of participants - Can allow the facilitator to engage private conversations with individual participants - Allows to build memory books (with pictures of the session) easily and instantaneously - For games, allows to easily share scores and personal progresses - Possibility to record patient activity, to improve follow-up and assessment of longitudinal changes - Possibility to easily record several “indirect” data (voice, gaze, movements, etc.)	- Time-consuming setup (for some devices) - Poor acceptability (and fear of not understanding) of the technology - Need of patients’ and staff’s training - Expensive equipment (e.g., VR headsets) - Absence of direct human contact (risk of reducing the opportunities of social interaction) - Homebound - Possibility of poor engagement/interest - Need to prepare the sessions in advance (technical calls to verify that participants can connect, etc.) - Need to send in advance materials to participants (e.g., odor sticks, tablets, etc.) - Difficulty to organize highly interactive activities (e.g., drawing together) - Dependence on internet availability - Need of individual support (caregiver on place)
**Opportunities**	**Threats**
- Emerging advances in technology - Good accessibility for users, also remotely (at home or in remote clinical facilities) - Increasing number of seniors commonly using ICT - Could help reducing barriers in access to care in middle- and low-income countries with limited access to specialized centers - Usable at large scale - Recordings allow to improve and standardize facilitators’ trainings - In case of positive experience, increased acceptability of ICT technologies - Prevention of isolation in the case of limitation of physical attendance (personal or general) - Indirect Follow-up of mental health in otherwise isolated population - Larger population recruiting enables more homogenous group selection, even in the case of rare phenotypes - Adaptability to sensory disabilities with specific group selections (e.g., visual and hearing difficulties).	- Low experience in ICT by users - Cognitive/behavioral fundamentals of the classical therapies are not fully reproduced - Not enough research evidence toward effectiveness, risk and impact. - Digital divide - Non-adapted ergonomics of several ICT tools - Increase of isolation of non-connected individuals - Decrease of individual activities and on-site workshops (financial threats) - Group Digital supports not adapted to individual sensory disabilities (size, contrast, sound)

#### Strengths

In terms of *organization*, remote workshops can help to connect people living in remote areas and/or with difficulties to commute, thus allowing more patients and elderly people access to care. This is particularly important when physical meetings are impossible, due for instance to health-related issues/restrictions, but also to traveling. Remote participation can limit the organizational constraints for workshops requiring long durations (several months) or frequency (everyday), being time and cost-effective (e.g., no need to commute). Remote workshops can facilitate interactions with the whole group, but also between couples of participants, and between each participant and the facilitator, using if needed personal chats and personal calls. Thanks to the recordings, it is easy to create screenshots of art works, game session results, pictures of participants, that can be used, after obtaining explicit approval from the participants, to create “storybooks” and provide feedback to participants easily and instantaneously.

In terms of patients’ *follow-up*, remote format gives the possibility to record patient activity, thus allowing facilitators to improve follow-up and the assessment of longitudinal changes. In addition, video-meetings can allow to easily record several “indirect” data (such as voice, gaze, and movements) that can also contribute to patient’s assessment over time.

#### Weaknesses

As all tools based on new technologies, setup can be time-consuming (at least for some devices), especially for people that are not familiar with ICT. Some people may not have available at home the materials they need (e.g., internet connection, laptop/tablet, camera to join with video, updated browsers), and some devices may be expensive and hard to set-up remotely. There is often the need to train patients, caregivers and facilitators to employ remote connection tools. Remote workshops need a more time-consuming preparation. For instance, it is necessary to perform technical calls before the actual workshop to verify that participants are able to connect correctly, that the video and sound quality is good, that there is not too much background noise, etc. Furthermore, if workshops rely on specific materials to be employed (e.g., painting materials, odor sticks, dices) these must be shipped to participants before the session. If participants are not autonomous with ICT, it is necessary that someone (a family caregiver, an assistant) is present to guarantee the connection. Also, it is harder compared to physical meetings to organize highly interactive activities (e.g., drawing together). Another weakness of remote workshops is that people that have a poor acceptability (and/or fear of not understanding) new technologies may be less willing to participate and/or less engaged in the activities. Remote workshops may reduce the amount of face-to-face human contact, with the risk of reducing the opportunities of social interactions, and increasing homebound.

#### Opportunities

Emerging advances in technology are improving device usability, accessibility and reducing costs of access, allowing more and more people to connect remotely. More and more houses and clinical facilities are equipped the video-conferencing materials, and more and more seniors today commonly employ new technologies such as smartphones, laptops and tablets. Remote workshops could help reducing barriers in access to care in middle- and low-income countries with limited access to specialized centers, and are usable at large scale. They may prevent isolation in the case of limitation of physical attendance (personal or general).

Sessions recordings may allow to improve and standardize facilitators’ trainings, thus potentially disseminating the use of good practices over different clinical facilities. In case of positive experience, there may be an increased acceptability of ICT technologies, allowing for a wider use. Larger population recruiting enables more homogenous group selection, even in the case of rare phenotypes. Finally, remote workshops may facilitate adaptability to sensory disabilities with specific groups (e.g., visual and hearing difficulties).

#### Threats

For remote workshops, it is necessary to adapt the format and materials. Due to this, cognitive/behavioral fundamentals of the classical therapies are not fully reproduced. Another threat is that, as the field is quite new, there is not enough research evidence toward effectiveness, risk and impact. Remote workshops may increase the sense of isolation of non-connected individuals due to digital divide. Furthermore, this may decrease the amount of individual activities and on-site workshops (financial threats). Finally, group digital supports mat not be adapted to individual sensory disabilities (size, contrast, and sound).

## Conclusion

The main objective of the present publication was to provide recommendations on the use of workshops with arts and board games, to orient clinicians in their daily practice, and to help researchers standardizing their procedures, thus potentially increasing reproducibility of practices and studies.

This work is in line with recent efforts to provide practical guidelines for non-pharmacological treatments in patients with Alzheimer’s disease and related disorders (see text footnote 7). Having group meetings in the same physical space is an innate human desire. However, the present recommendations also focus on remote/hybrid workshops, whose use may become more and more common. It is important that this type of practice can be used while respecting at the same time a standardization while preserving as much as possible the freedom of the participants.

## Data Availability Statement

The raw data supporting the conclusions of this article will be made available by the authors, without undue reservation.

## Ethics Statement

Ethical review and approval was not required for the study on human participants in accordance with the local legislation and institutional requirements. The patients/participants provided their written informed consent to participate in this study.

## Author Contributions

VM and PR designed the study, facilitated the Delphi procedure. VM and RF performed the data analyses. All authors participated to the Delphi panel and wrote the manuscript.

## Conflict of Interest

PF was employed by the company GENIOUS Healthcare–Mindmaze Group Co. ML and LM were employed by the company Asmodee Research Co. PM was employed by the company NEHS Digital Co. The remaining authors declare that the research was conducted in the absence of any commercial or financial relationships that could be construed as a potential conflict of interest.

## Publisher’s Note

All claims expressed in this article are solely those of the authors and do not necessarily represent those of their affiliated organizations, or those of the publisher, the editors and the reviewers. Any product that may be evaluated in this article, or claim that may be made by its manufacturer, is not guaranteed or endorsed by the publisher.

## Footnotes

1 https://univ-cotedazur.fr/formation-continue/acces-e-learning https://www.education.gouv.fr/continuite-pedagogique-faciliter-le-travail-distance-avec-appseducationfr-303738

2 https://www.msa.fr/lfy/sante/ateliers-prevention-retraite https://www.lassuranceretraite.fr/portail-info/sites/pub/hors-menu/annexe/retraites/offres-digitales-accessibles-et.html

3
http://www.innovation-alzheimer.fr/art-sante/

4
https://www.game-in-lab.org/projets/projet-cab/

5 Link to be added

6
https://www.who.int/news-room/fact-sheets/detail/mental-health-of-older-adults

7
https://www.fondation-mederic-alzheimer.org/sites/default/files/2021_guide_interventions_non_medicamenteuses.pdf

8
https://www.sense-cog.eu/the-project/index.html
